# Supplementary material for: BioTEA: Containerized Methods of Analysis for Microarray-Based Transcriptomics Data
Source: Biology (Basel). 2022 Sep 13;11(9):1346. doi: 10.3390/biology11091346 (PMC9495986; doi:10.3390/biology11091346)
Supplement: Supplementary file 1 [file biology-11-01346-s001.zip › bioTEA_Supplementary.pdf]

BioTEA: containerized methods of analysis for microarray-based transcriptomics data

Visentin L.[1], Scarpellino G.[1], Chinigò G.[1], Munaron L.[1], and Ruffinatti F. A.[1]

1: Department of Life Sciences and Systems Biology, University of Turin, Turin 10123, Italy

Supplementary Methods

File S1. Options of the analyze module

The `biotea analyze` options are divided into sections. Each section controls different aspects of the analysis. The possible types of the various options are annotated in parentheses. The available options are:

- **`general`**: The general section contains general options to control plot size and type, whether to include annotations, and if to print extra data snippets during the analysis:
  - **`show_data_snippets` (`bool`)**: If `true`, prints extra snippets of data during the analysis. This is useful to see if the analysis is doing anything wrong. The default is `true`.
  - **`annotation_database` (`bool` or `str`)**: This value controls how the data is annotated. Annotating data allows for plots with gene names instead of probe IDs, and a more useful output. If `true`, the data is annotated with an internal annotation file covering most Agilent and Affymetrix Human chips. If `false`, the data is not annotated. Otherwise, a string can be passed, representing the complete name of any annotation package on Bioconductor, which will be downloaded and installed on the fly, and used to source annotations. Note: the package must contain annotations for `SYMBOL`s, `ENSEMBL` IDs, and `GENENAME`s, or the annotation will fail. This might be addressed in a future version of bioTEA.
  - **`plots`**: Various options to control plots:
    - **`save_png` (`bool`)**: If `true`, saves plots in `.png` format. Otherwise, saves in `.pdf` format.
    - **`plot_width` (`int`)**: The width of the plots, in inches.
    - **`plot_height` (`int`)**: The height of the plots, in inches.
    - **`png_resolution` (`int`)**: The pixels per inch of the png plots.
    - **`enumerate_plots` (`bool`)**: If `true`, each plot is marked with a number, in the order it is created.
- **`switches`**: The switches section contains various parameters to turn parts of the analysis on or off:
  - **`dryrun` (`bool`)**: Run the analysis, but do not save any output plots (with the exception of the log file). This can be useful to test out the analysis before committing to it, especially when used in combination with the `show_data_snippets` option.
  - **`renormalize` (`bool`)**: Run quantile-quantile normalization on the data. This can be useful to normalize "unruly" samples, for which the normalization steps in `prepare affymetrix` and `prepare agilent` are not enough. Additional plots are saved to appreciate this extra normalization step.
  - **`limma` (`bool`)**: Run DEA with `limma`?
  - **`rankproduct` (`bool`)**: Run DEA with `RankProduct`?
  - **`convert_counts`**: If the input data is count data (e.g. RNAseq data), set this to `true` to use `voom` to transform the data to continuous values before the analysis. Count values by themselves are unsupported. Defaults to `false`.
- **`design`**: The design section contains crucial options to set the experimental design that will be used to steer the analysis.
  - **`experimental_design` (`str`)**: The experimental design of the experiment. It

must be a comma-delimited set of values, of the same length as the number of input samples, with each value being the label for the experimental variable of interest. Numbers specified at the end of each group can be used to represent sample pairings. Shorthands are supported: `(a, b):2` is expanded to `a, b, a, b`, and `[a, b]:2` in expanded to `a, a, b, b`. This allows for fast inputation of the design string even for large datasets. Shorthands are also available to specify sample pairings. For more information, consult the online guide.

- **`contrasts` (`list of str`)**: A list of strings of the type `"group1-group2"`, where each "group" is a level in the experimental design. Each value in the list specifies a (different) contrast of interest. The second group in each contrast is considered the "background" or "control" status (i.e. "group2"). For more information, consult the online guide.
- **`batches` (`null` OR `str`)**: If `null`, no batch effect will be corrected, assuming all samples derive from the same batch. If `str`, it is treated similar to the `experimental_design` string, and each level refers to a different batch. Note that `RankProd` cannot correct batch effects if, inside each batch, there are not at least than two samples for each experimental condition.
- **`extra_limma_vars` (`null` OR nested `str`**: If `null`, nothing happens. If nested `str`, each string in the nested list is treated similar to the `experimental_design` string, adding an additional variable to the `limma` analysis. This can be useful to control for additional confounding variables in the experiment. For an example, see the default configuration file itself.
- **`group_colors` (`list of str`)**: A list of strings that can be understood by `R` to be a colour. Each colour will be paired with a different condition type in the experimental design, so at least that many colours must be specified.
- **`filters`**: The parameters used by the analysis to filter the data:
  * **`log2_expression` (`float`)**: Filter out any genes that have lower Log2 expression than this value. This value depends a lot on the experiment, and is generally higher for Agilent arrays. The default of "4" is good for Affymetrix arrays.
  * **`fold_change` (`float`)**: Filter out (mark as non-differentially-expressed) any genes that have lower absolute Fold Change than this value. This is used to remove from the analysis all genes that, even if detected to be a DEG, as additional validation with other methods (such as `PCR`) would be impossible.
  * **`min_groupwise_presence` (`float`)**: A value between 0 and 1, representing the proportion of samples in a single group of interest in which the `fold_change` filter threshold must be violated to be filtered out. If a gene passes the filter in at least one group, it is retained in the analysis. This allows for a more conservative filtering of the genes.

This information is accurate for the version of bioTEA at the moment of writing (`v.1.0.1`). For updates, please refer to the online repository.

File S2. Commands used for the Zhang Example Analysis

To reproduce the Zhang analysis, follow these instructions:

1. Install bioTEA as indicated on GitHub and the main text and `gunzip` (or equivalent).
2. In a working directory of choice (possibly empty), hereby referred to as `<WD>`, use `biotea retrieve <WD> GSE28735` to retrieve the Zhang data from GEO. The `FTP` protocol has to be available.
3. Prepare the data by first unzipping the input data in `<WD>/unpacked_samples` with `gunzip <WD>/unpacked_samples`.
4. Run `biotea prepare affymetrix --version "1.0.1" --remove-controls <WD>/unpacked_samples/ <WD>/expression_matrix.csv` to generate the expression matrix. The appropriate docker container is automatically downloaded if missing.
5. Download the `bioTEA_run_options.yaml` files from the supplementary materials. Save it in `<WD>`.
6. Run the analysis with `biotea analyze --version "1.0.1" <WD>/bioTEA_run_options.yaml <WD>/analysis_results <WD>/expression_matrix.scv`.

The analysis results are generated and saved in `<WD>/analysis_results`.
